# Advancing bovine *in vitro* fertilization through 3D printing: the effect of the 3D printed materials

**DOI:** 10.3389/fbioe.2023.1260886

**Published:** 2023-10-19

**Authors:** Ramses Belda-Perez, Sonia Heras, Costanza Cimini, Jon Romero-Aguirregomezcorta, Luca Valbonetti, Alessia Colosimo, Bianca Maria Colosimo, Silvia Santoni, Barbara Barboni, Nicola Bernabò, Pilar Coy

**Affiliations:** ^1^ Department of Biosciences and Technology for Food, Agriculture and Environment, University of Teramo, Teramo, Italy; ^2^ Physiology of Reproduction Group, Department of Physiology, Faculty of Veterinary Medicine, International Excellence Campus for Higher Education and Research (Campus Mare Nostrum), University of Murcia, Murcia, Spain; ^3^ Institute of Biochemistry and Cell Biology (CNRIBBC/EMMA/Infrafrontier/IMPC), National Research Council, Rome, Italy; ^4^ Department of Mechanical Engineering, Politecnico di Milano, Milano, Italy

**Keywords:** IVF, bovine, embryo culture, biomaterials, PCL, PEGDA, PLA

## Abstract

Nowadays there is an increasing demand for assisted reproductive technologies due to the growth of infertility problems. Naturally, fertilization occurs in the oviduct, where the oviductal epithelial cells (OECs) secrete many molecules that affect the embryo’s metabolism and protect it from oxidative stress. When the OECs are grown in 3D culture systems, they maintain a great part of their functional characteristics, making them an excellent model for *in vitro* fertilization (IVF) studies. In this work, we aimed to evaluate the suitability of different 3D-printing processes in conjunction with the corresponding set of commercially available biomaterials: extrusion-based processing using polylactic acid (PLA) and polycaprolactone (PCL) and stereolithography or digital-light processing using polyethylene-glycol-diacrylate (PEGDA) with different stiffness (PEGDA500, PEGDA200, PEGDA PhotoInk). All the 3D-printed scaffolds were used to support IVF process in a bovine embryo assay. Following fertilization, embryo development and quality were assessed in terms of cleavage, blastocyst rate at days 7 and 8, total cell number (TCN), inner cell mass/trophectoderm ratio (ICN/TE), and apoptotic cell ratio (ACR). We found a detrimental effect on cleavage and blastocyst rates when the IVF was performed on any medium conditioned by most of the materials available for digital-light processing (PEGDA200, PEGDA500). The observed negative effect could be possibly due to some leaked compound used to print and stabilize the scaffolds, which was not so evident however with PEGDA PhotoInk. On the other hand, all the extrusion-based processable materials did not cause any detrimental effect on cleavage or blastocyst rates. The principal component analysis reveals that embryos produced in presence of 3D-printed scaffolds produced via extrusion exhibit the highest similarity with the control embryos considering cleavage, blastocyst rates, TCN, ICN/TE and ACR per embryo. Conversely, all the photo-cross linkable materials or medium conditioned by PLA, lead to the highest dissimilarities. Since the use of PCL scaffolds, as well as its conditioned medium, bring to embryos that are more similar to the control group. Our results suggest that extrusion-based 3D printing of PCL could be the best option to be used for new IVF devices, possibly including the support of OECs, to enhance bovine embryo development.

## 1 Introduction

In recent years, the demand for artificial reproductive technologies (ARTs) is growing due to an increase in infertility, which already affects 15% of couples of reproductive age and continues to rise every year (Assidi, 2022). The high number of infertile couples, together with reproductively healthy ones seeking to prevent genetic diseases in their offspring, have contributed to an increase in the proportion of children born through ARTs in Europe, from 2.3% (De Geyter et al., 2020) to 3.5% (Gliozheni et al., 2022) in just 3 years. In human reproduction, a popular technique is intracytoplasmic sperm microinjection (ICSI) (Haddad et al., 2021), in which a sperm selected by the embryologist is directly injected into the ooplasm. With this technique, positive results are obtained despite the low motility of the sample or immaturity of the sperm (Palermo et al., 1996). However, there are main concerns about ICSI for its invasiveness since it involves the piercing of the membrane. As a result, it could induce spindle damage or the introduction of contaminating external material (Verpoest and Tournaye, 2009). Another option is *in vitro* fertilization (IVF), where the oocyte and sperm are co-cultured in the same plate for a certain period so that penetration occurs without human intervention. Although IVF has been associated with an increased risk of congenital diseases or developmental delay (Waynforth, 2018), this method is considered the most physiological, since the spermatozoa penetrates the oocyte by itself. In addition, the scientific community is increasingly concerned about the potential long-term effects of ARTs (Sunde et al., 2016; Fleming et al., 2018). It is known that suboptimal *in vitro* conditions influence the epigenetic reprogramming of embryos (Canovas et al., 2017; Ferraz et al., 2018b). In humans, it has been suggested that ARTs may be related to a higher risk of imprinting disorders such as Beckwith-Wiedemann (Maher et al., 2003) or Angelman syndrome (Manipalviratn et al., 2009), although in the latter, it is very difficult to understand whether these disorders are related to the couple´s infertility-subfertility problems or to ARTs (Pérez-Aytés et al., 2017). Moreover, differences in growth in ARTs-derived offspring in pig (París-Oller et al., 2022) and human (Ceelen et al., 2009) have also been observed.

All these above-mentioned problems could be solved by mimicking the physiological environment (i.e., the oviduct). In this organ, the oviductal epithelial cells (OECs) produce a large number of molecules that can protect embryos from oxidative stress and modify their metabolism (Ménézo et al., 2015). Indeed, two alternative strategies can be used to replicate natural conditions: 1) the use of reproductive fluid as a culture media supplement (Canovas et al., 2017) and 2) co-culture of gametes and embryos with oviductal epithelial cells (OECs) (Ferraz et al., 2018b). Two-D cultures (where cells grow in a monolayer) are the most popular for studying the physiology of the oviduct and have been used in IVF and embryo culture in several species (Kölle et al., 2020), probably due to their high reproducibility, low cost, or ease of handling (Costa et al., 2016). Indeed, when these cultures are used during embryo *in vitro* production (IVP), there is an enhanced developmental rate of bovine embryos (Abe and Hoshi, 1997). However, it has been shown that this 2D culture method is not the best suited for fertilization studies since the cells dedifferentiate and lose their polarity, morphology, secretory capacity, and ciliary activity (Ferraz et al., 2017). On the contrary, when cultured in 3D, these cells retain much of their natural features (Pennarossa et al., 2021), making them a better model by keeping gene and metabolic expression closer to the *in vivo* context than their 2D counterparts (Anton et al., 2015). When the physiological environment is mimicked using microfluidics culture during IVF, it has been shown that the epigenetic reprogramming of bovine embryos is more similar to *in vivo* derived embryos (Ferraz et al., 2018b). All these data are indicators of the limitations of the 2D culture methods, thus encouraging researchers to move towards 3D culture systems to improve the quality of IVP embryos. To achieve this goal, it is crucial to construct a 3D device in which it is possible to co-culture differentiated OECs with gametes/zygotes. As a matter of fact, despite the well-known relevance the oviduct in gamete maturation/activation, fertilization, and early embryo development, only a few bioengineering studies have been focused on these female reproductive structures, so far (Kessler et al., 2015; Xiao et al., 2017; Ferraz et al., 2018b; Ferraz et al., 2020; Francés-Herrero et al., 2022).

Nowadays, a great variety of 3D printable biomaterials are commercially available (Santoni et al., 2021). One popular biomaterial is polylactic acid (PLA), a promising biodegradable polymer that can be produced from renewable sources like sugarcane (Li et al., 2020). PLA-scaffolds have excellent biocompatibility (Shilov et al., 2022), and have been used for medical purposes in bone (Diomede et al., 2018; Velioglu et al., 2019) and cartilage regeneration (Rosenzweig et al., 2015). Together with PLA, polycaprolactone (PCL) is the most common biodegradable synthetic polymer used in tissue engineering (Arif et al., 2022), and it has already been employed for bone (Rumiński et al., 2018), liver (Huang et al., 2007) or skin (Ghosal et al., 2017) regenerative purposes. Similarly, photo-cross-linkable hydrogels are widely used, due to their tunable mechanical properties and to their capability to mimic native extracellular matrix (Lim et al., 2020; Zhang et al., 2022a). In fact, when viscoelasticity and stiffness properties of biomaterials can be tuned, this can represent an additional advantage to create scaffolds mimicking the native tissues with high resolution and complex architecture. Among them, polyethylene-glycol-diacrylate (PEGDA) is a synthetic polymer approved by the Food and Drug Administration (FDA) (Kim et al., 2022) that has been used in variegate studies, from bone (Rajabi et al., 2023) to cartilage (Zhang et al., 2022b) or muscle (Vannozzi et al., 2018) regeneration. In addition, PEGDA mechanical properties can be modulated by varying the molecular weight of the polymer (Nguyen et al., 2012) and it can be functionalized with cell binding motifs to enable cell adhesion (Della Sala et al., 2020). Despite the wide range of biomedical applications in which these materials have been used, no studies have been carried out so far to test the feasibility of these materials to construct a 3D-printed device for IVF.

Because of this lack of information, our study aims to evaluate the biocompatibility of different materials (PLA, PCL, PEGDA500, PEGDA200, and PEGDA PhotoInk) to support IVF, using bovine embryo development parameters (cleavage, blastocyst rates at day 7 and 8). In addition, to assess the quality of the *in vitro* produced embryos, we examined three fundamental parameters (Wydooghe et al., 2014) the cell number/embryo (TCN), the inner cell mass/trophectoderm (ICM/TE) ratio, and the apoptotic cell ratio (ACR).

## 2 Materials and methods

### 2.1 Experimental design

**FIGURE 1:**
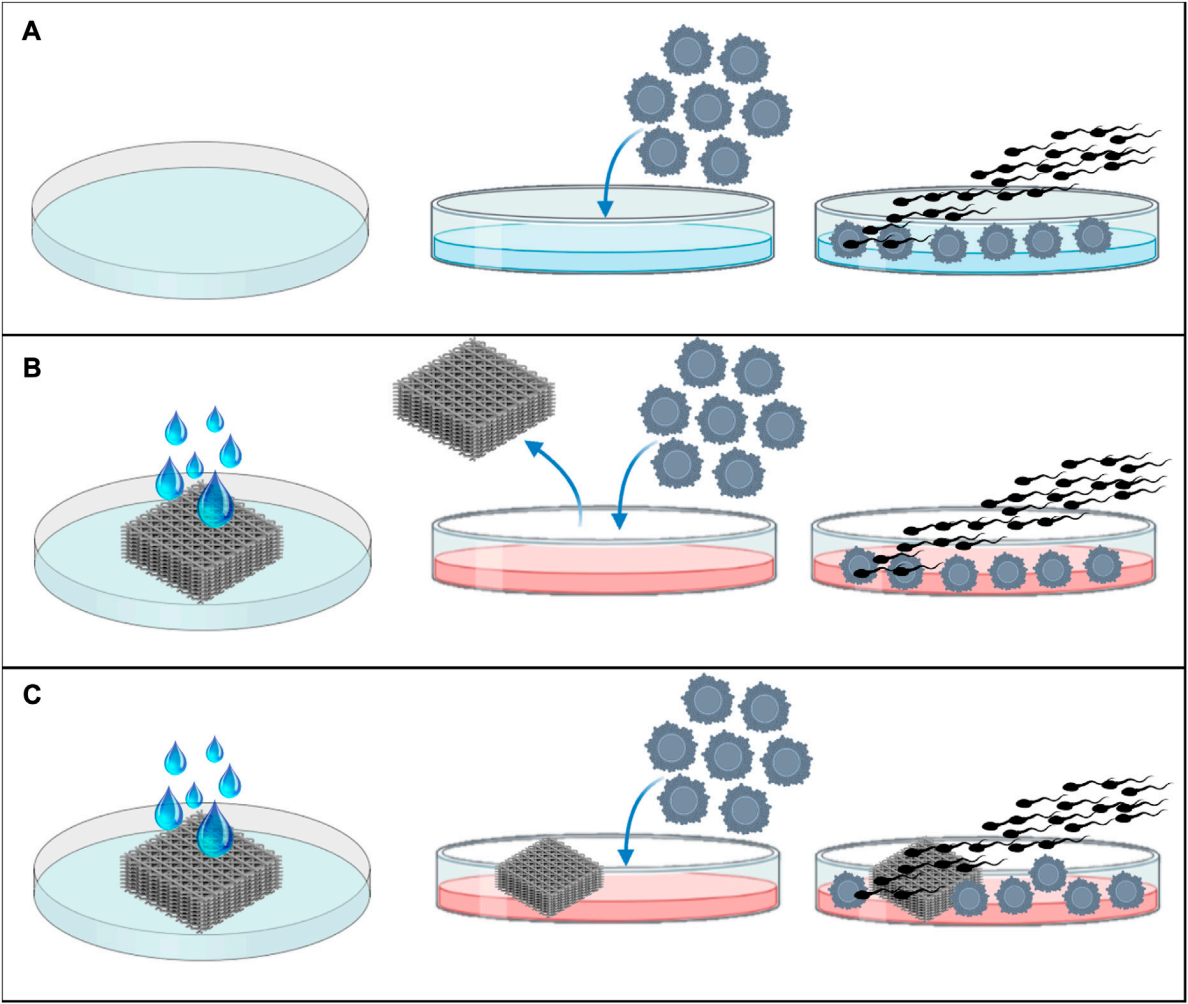
Experimental design. **(A)** IVF in normal Fert-TALP medium, without conditioning or scaffold, **(B)** IVF in Fert-TALP medium conditioned by scaffold for 24 h, and **(C)** IVF performed in presence of the rinsed scaffold.

To evaluate the feasibility of different materials (PLA, PCL, PEGDA500, PEGDA200, and PEGDA PhotoInk) to support IVF and their effects on bovine embryo development, three experimental groups were settled for each of the materials tested:

- Control group: the IVF was performed, without having any contact with the materials (Figure 1A).
- Rinse group: to assess if these materials could release some unknown substances that could have adverse effects in IVF or embryo development, the IVF was carried out in a Fert-TALP medium conditioned by the scaffold of each material during 24 h (Figure 1B).
- Scaffold group: prior to fertilization, the same scaffold used to condition the IVF media, was transferred to another well with new media and the IVF was performed in the presence of the scaffold (Figure 1C).

The number of oocytes was *n* = 2892 distributed for each group as follows: *n* = 585 for control, *n* = 297 for scaffold PCL, *n* = 293 for rinse PCL, *n* = 196 for scaffold PLA, *n* = 201 for rinse PLA, *n* = 131 for scaffold PEGDA500, *n* = 143 for rinse PEGDA500, *n* = 248 for scaffold PEGDA200, *n* = 256 for rinse PEGDA200, *n* = 269 for scaffold PEGDA PhotoInk, *n* = 273 for rinse PEGDA PhotoInk. We carried out 11 replicates, the control group was present in every single replicate, while PCL material was present in 6 of them, the PLA material in 4, the PEGDA500 material in 3, and the PEGDA200 and PEGDA PhotoInk materials were present in 5 replicates.

### 2.2 Culture media reagents.

All chemicals were purchased from Sigma-Aldrich Quimica, S.A. (Madrid, Spain) unless otherwise indicated.

### 2.3 3D printing materials

3D printed structures were produced to test the biocompatibility of the material and the effect of the 3D architecture on cells. All structures were designed using SolidWorks software (Dassault Systèmes SE, Vélizy-Villacoublay, France) and exported as an STL file. Depending on the printer used, the STL file was directly loaded on the printer or sliced using PrusaSlicer (Prusa Research, Prague, Czech Republic) to obtain the gcode file.

The 3D models were printed using different materials with different stiffness (Table 1) and 3D printing methods. PLA filaments were purchased from Sharebot, Italy; PCL pellet (Mn = 50.000 g/mol), PEGDA200, PEGDA500, and PEGDA Photoink were purchased from Cellink, Sweeden. PLA filaments were printed via extrusion-based processing (FFF, Fused Filament Fabrication) using a Sharebot 42 3D printer (Sharebot, Italy) with a 0.4 mm diameter nozzle. PCL (CELLINK, Gothenburg, Sweden) structures were 3D printed using a BioX, a pneumatic extrusion-based 3D bioprinter (CELLINK, Gothenburg, Sweden) using a 0.4 mm nozzle, a pressure of 180 kPa, a velocity in a range 15–20 mm/s, and a temperature of 180°C according to suggested printing protocol. PEGDA500 Photoink, PEGDA200 Photoink, and PEGDA Photoink hydrogels (listed in decreasing order of stiffness) were 3D printed using a LumenX bioprinter based on stereolithography via digital light processing (CELLINK, Gothenburg, Sweden) considering a 50 µm layer height for the slicing and 20 mWatt/cm^2^ power, 3x as first layer time scale factor, and a variable time of 2/3/12 s depending on the formulation, respectively, according to printing protocol.

**TABLE 1:** Hardness of different materials (PLA, PCL, PEGDA500, PEGDA200, and PEGDA PhotoInk) expressed by Young’s modulus.

Material	Young modulus	Source
PLA	3,000 MPa	Manufacturer (Sharebot)
PCL	370 MPa	Scocozza et al. (2023)
PEGDA500	500 KPa	Manufacturer (Cellink)
PEGDA200	200 KPa	Manufacturer (Cellink)
PEGDA PhotoInk	50 KPa	Manufacturer (Cellink)

### 2.4 Material sterilization

PLA, PCL, PEGDA500, PEGDA200, and PEGDA PhotoInk were sterilized following the manufacturer’s instructions. Briefly, they were immersed in 70% ethanol for 5 min, then submerged twice in PBS (30 min each), and finally washed for 24 h with Fert-TALP (Parrish et al., 1986) culture medium supplemented con 175 U/mL heparin, 6 mg/mL BSA, 0.20 mM Na-pyruvate and 50 μg/mL gentamicin. Fert-TALP medium consisted of 114 mM sodium chloride, 3.2 mM potassium chloride, 0.3 Mm sodium phosphate monobasic monohydrate, 10 mM sodium lactate, 2.0 mM calcium chloride dihydrate, 0.5 mM magnesium chloride hexahydrate and 25 mM sodium bicarbonate.

### 2.5 *In vitro* maturation

Ovaries from 1 year old cows were transported from the local slaughterhouse to the laboratory in physiological saline solution (0.9% w/vol) supplemented with 100 mg/L kanamycin sulfate at 38.5°C within two hours of slaughter. Once in the laboratory, the ovaries were washed with a 0.04% cetrimide solution and twice with saline. *In vitro* maturation was performed as previously described (Lopes et al., 2019) with minor modifications. Briefly, follicles between 2- and 8-mm diameter were aspirated. Only Cumulus-Oocyte Complexes (COCs) with at least three cumulus cell layers and with a homogeneous cytoplasm were selected and then washed three times in handling medium, consisting of TCM 199 supplemented with 4.2 mM sodium bicarbonate, 10 mM HEPES, 2 mM glutamine, 1% w/v polyvinyl alcohol, 50 IU/mL penicillin and 50 μg/mL streptomycin. Subsequently, COCs were washed once in a maturation medium, consisting of TCM 199 (with Hanks’ salts) supplemented with 4.2 mM sodium bicarbonate, 2 mM glutamine, 50 IU/mL gentamicin, 10% v/v of bovine follicular fluid (BFF, NaturARTs-BFF, Embryocloud, Murcia, Spain), 10 IU/mL equine chorionic gonadotropin (Foligon, Intervet International BV, Netherlands) and 10 IU/mL human chorionic gonadotropin (Veterin Corion, Divasa Farmavic, Spain) and incubated in 500 μL of maturation medium in groups of 50–55 COCs in a four well dish at 38.5°C with a humidity-saturated atmosphere with 5% CO_2_ for 22 h.

### 2.6 *In vitro* fertilization

After maturation and 30 min before IVF, the oocytes were washed once in Fert-TALP medium supplemented con 175 U/mL heparin, 6 mg/mL BSA, 0.20 mM Na-pyruvate and 50 μg/mL gentamicin. For fertilization, frozen-thawed semen from three bulls of proven fertility was used. The straw was thawed in a water bath at 38.5°C for 30 s. Once thawed, a Bovipure gradient (Nidacon, Sweden) was performed, centrifuging at 300 g for 10 min and removing the supernatant. Before insemination, sperm cells were washed once in modified Sperm-TALP medium (Parrish et al., 1988) (114 mM sodium chloride, 3.2 mM potassium chloride, 0.3 Mm sodium phosphate monobasic monohydrate, 10 mM sodium lactate, 2.0 mM calcium chloride dihydrate, 0.5 mM magnesium chloride hexahydrate, 25 mM sodium bicarbonate and 10 mM HEPES) supplemented with 6 mg/mL BSA, 1.0 mM Na-pyruvate and 50 μg/mL gentamicin, by centrifuging at 300 g during 3 min and removing the supernatant. Insemination was performed in medium conditioned by the scaffold, in the presence of scaffold and in fresh medium, in a final concentration of 1 × 10^6^ spz/mL. Oocytes were coincubated with the spermatozoa for 22 h at 38.5°C with a humidity-saturated atmosphere with 5% CO_2._

### 2.7 Embryo culture

Twenty-two hours after insemination, the presumptive zygotes were moved into a 15 mL Falcon tube with a handling medium and vortexed for 4 min for decumulation. Zygotes were then washed once in Synthetic Oviductal Fluid medium (SOF) (Holm et al., 1999) and transferred into 50 μL microdrops of the same media covered by paraffin oil (Nidoil, Nidacon) in groups of 25 embryos per drop and cultured during 8 days at 38.5°C, 5% CO_2_ and 5% O_2_. Evaluation of embryo development occurred 48 h post insemination as the percentage of cleaved embryos out of presumptive zygotes, and at 7 and 8 days post insemination (dpi). In this study, only embryos with quality 1–2 according to the criteria of the International Embryo Technology Society (IETS) (summarized in Bó and Mapletoft, 2013) have been considered.

### 2.8 Differential apoptotic staining

To assess the total cell number (TCN), the inner cell mass/trophectoderm ratio (ICM/TE), and the apoptotic cell ratio (ACR), differential staining was performed as described previously (Wydooghe et al., 2011) with minor modifications. Briefly, day 8 blastocysts were fixed in 4% paraformaldehyde for 20 min at RT and conserved in 2% paraformaldehyde at 4°C until the moment of staining. The embryos were permeabilized with 0.5% Triton-X and 0.05% Tween in PBS overnight at 4°C. On the second day, blastocysts were washed three times for 10 min in PBS containing 0.5% BSA (washing solution). Subsequently, the DNA of the cells was denatured with 2N HCl for 20 min followed by 100 Mm trisHCL (pH = 8.5) for 10 min. After denaturation, the embryos were washed three times in washing solution and transferred to blocking solution (10% goat serum and 0.05% tween in PBS) overnight at 4°C. After blocking, the blastocysts were washed three times in washing solution and incubated in ready-to-use mouse anti-CDX2 primary antibody (Biogenex, San Ramon, United States) for overnight at 4°C, while two embryos remained in blocking solution as negative control. After this incubation, test embryos were washed three times in washing solution and incubated 1:500 dilution of rabbit anti -active caspase-3 primary antibody (Cell Signaling Technology, Leiden, Netherlands) in blocking solution overnight at 4°C. On the last day, all blastocysts (negative and test) were washed three times for 10 min in washing solution, and incubated with 1:100 goat anti-mouse TRICT (Abcam, Cambridge, United Kingdom) in blocking solution for 1 h at RT. After another three-wash step, the embryos were incubated in 1:200 goat anti-rabbit FITC secondary antibody (Abcam, Cambridge, United Kingdom) in blocking solution for 1 h at RT. Finally, the blastocysts were washed, stained with Hoechst 33342 for 15 min, washed for the last time, mounted in Dabco (1,4-Diazabicyclo[2.2.2]octane solution), and evaluated under fluorescence microscopy (Eclipse Ti Series, Nikon, Japan). A representative image of embryo was taken using Nikon A1r laser confocal scanning microscope.

### 2.9 Statistical analysis

For statistical analysis, GraphPad Prism 8 Software (La Jolla, CA, United States) was used. Data were checked for normal distribution with Shapiro-Wilk normality test prior to perform the comparison with parametric tests. In all cases the differences among groups were considered statistically significant when *p* < 0.05.

For Principal Component Analysis, Past 4.13 (Oslo, Norway) was used to evaluate the effect of different materials on Cleavage, blastocyst rate at day 7, blastocyst rate at day 8, TCN, ICM/TE and AC ratio.

## 3 Results

### 3.1 Effect of the different materials on cleavage

**FIGURE 2:**
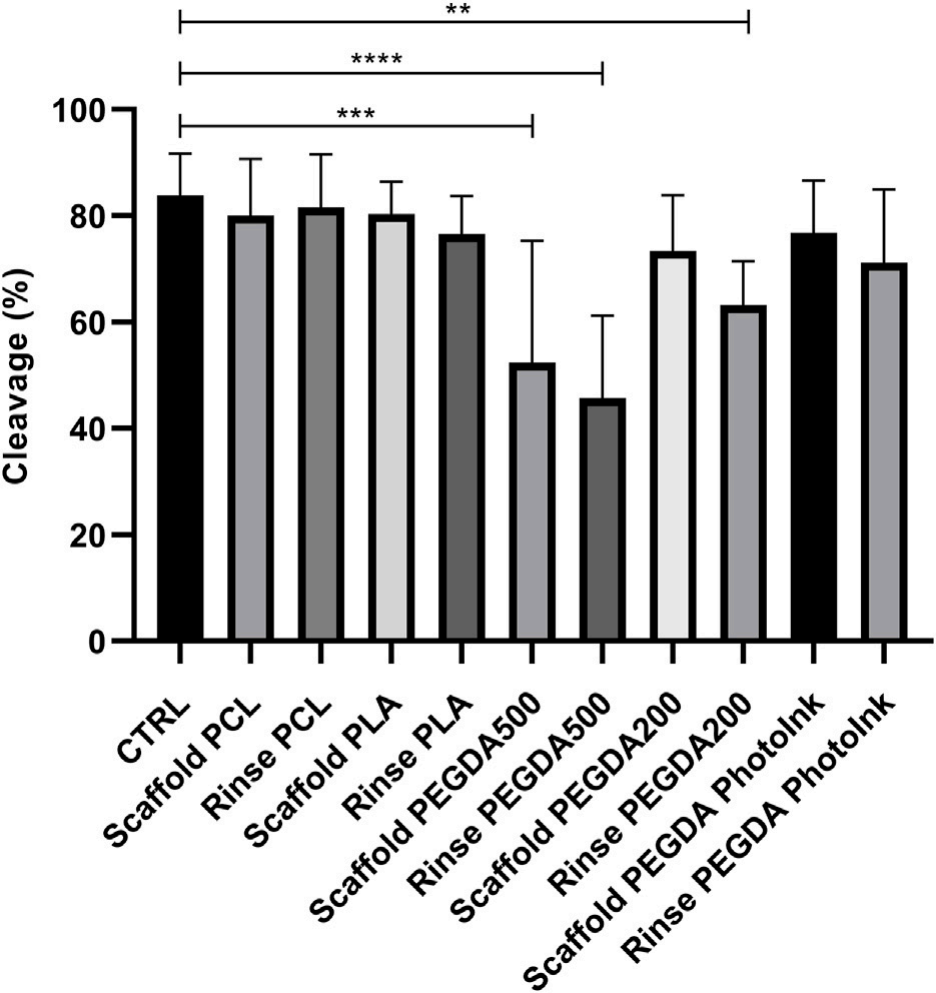
Effect of different materials on the percentage of cleaved embryos. The histograms show the cleavage rate when the IVF was performed with an unconditioned medium (CTRL), with medium conditioned by each material (rinse groups) or when different scaffolds were present (scaffold groups). The data show significant differences (*p* < 0.05) in CTRL vs. Scaffold PEGDA500, CTRL vs. Rinse PEGDA500 and CTRL vs. Rinse PEGDA200. The data are presented as the mean ± SD of 11 independent experiments. Data were analyzed using Dunnett’s test. **p < 0.01, ***p < 0.005, ****p < 0.0001 versus control.

We observed a significant lower cleavage rate in rinse PEGDA500 (45% ± 15%), scaffold PEGDA500 (50% ± 23%), and rinse PEGDA200 (63% ± 8%) groups vs. CTRL group (84% ± 8%), while we did not observe any difference between the cleavage rate of rinse PCL (82% ± 10%), scaffold PCL (80% ± 10%), rinse PLA (77% ± 7%), scaffold PLA (81% ± 6%), scaffold PEGDA200 (73% ± 11%), rinse PEGDA PhotoInk (71% ± 14%) or scaffold PEGDA PhotoInk (77% ± 10%) compared to the control (Figure 2).

### 3.2 Effect of the different materials on blastocyst rate at day 7

**FIGURE 3:**
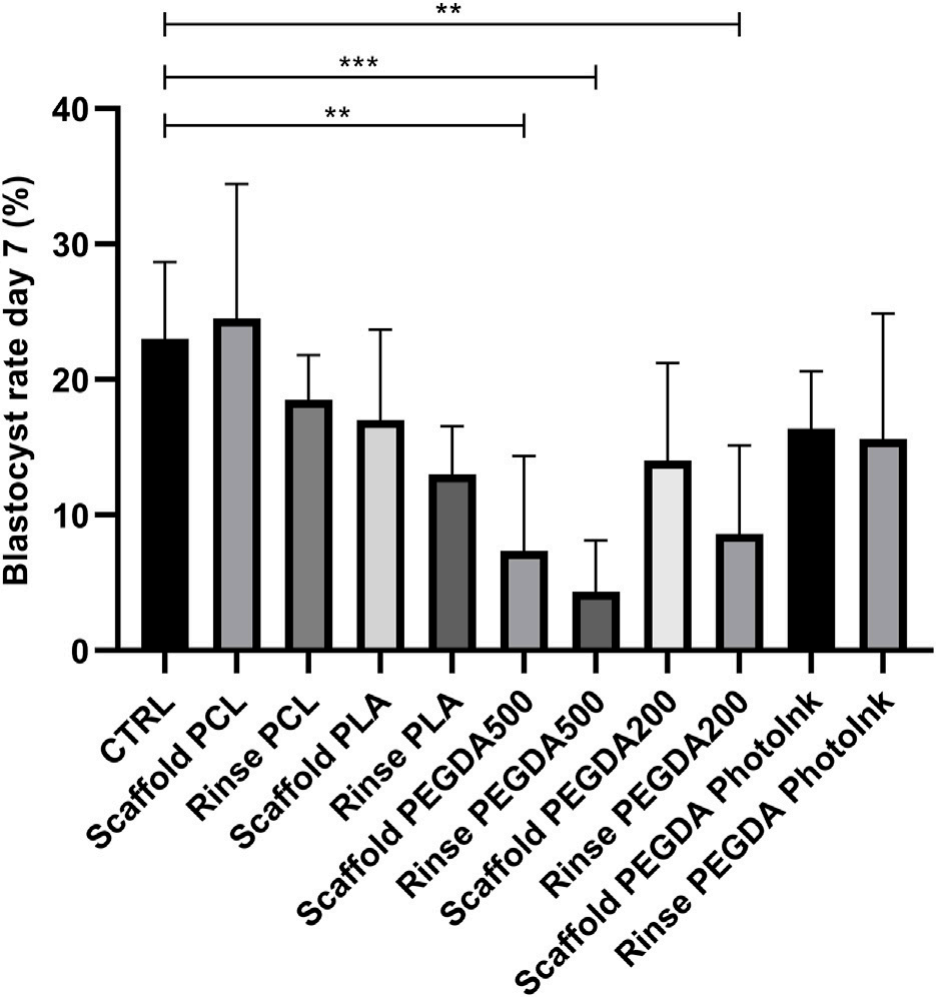
Effect of different materials on blastocyst rate at day 7. The histograms show the blastocyst rate at day 7 when the IVF was performed with unconditioned medium (CTRL), with medium conditioned by each material (rinse groups) or when different scaffolds were present (scaffold groups). The data show significant differences (*p* < 0.05) in CTRL vs. Scaffold PEGDA500, CTRL vs. Rinse PEGDA500 and CTRL vs. Rinse PEGDA200. The data are presented as the mean of 11 independent experiments. Data were analyzed using the Dunnett’s test. **p < 0.01, ***p < 0.005 versus control.

Compared to the CTRL group (23% ± 6%), blastocyst rates were significantly lower (*p* < 0.05) in the rinse PEGDA500 (4% ± 4%), scaffold PEGDA500 (7% ± 7%), and rinse PEGDA200 (8% ± 7%) groups on day 7 (Figure 3). The scaffold PCL group had a 25% ± 10% blastocyst yield, which was not statistically different (*p* > 0.05) vs. the CTRL, while scaffold PLA (17% ± 7%), scaffold PEGDA200 (14% ± 7%), and scaffold PEGDA PhotoInk (16% ± 4%) showed similar blastocyst rates. On the other hand, rinse groups have decreased embryo development compared to control but not significantly less than their scaffold groups, being 18% ± 4% for rinse PCL, 13% ± 4% for rinse PLA, and 16% ± 7% for rinse PEGDA PhotoInk (Figure 3).

### 3.3 Effect of the different materials on blastocyst rate at day 8

**FIGURE 4:**
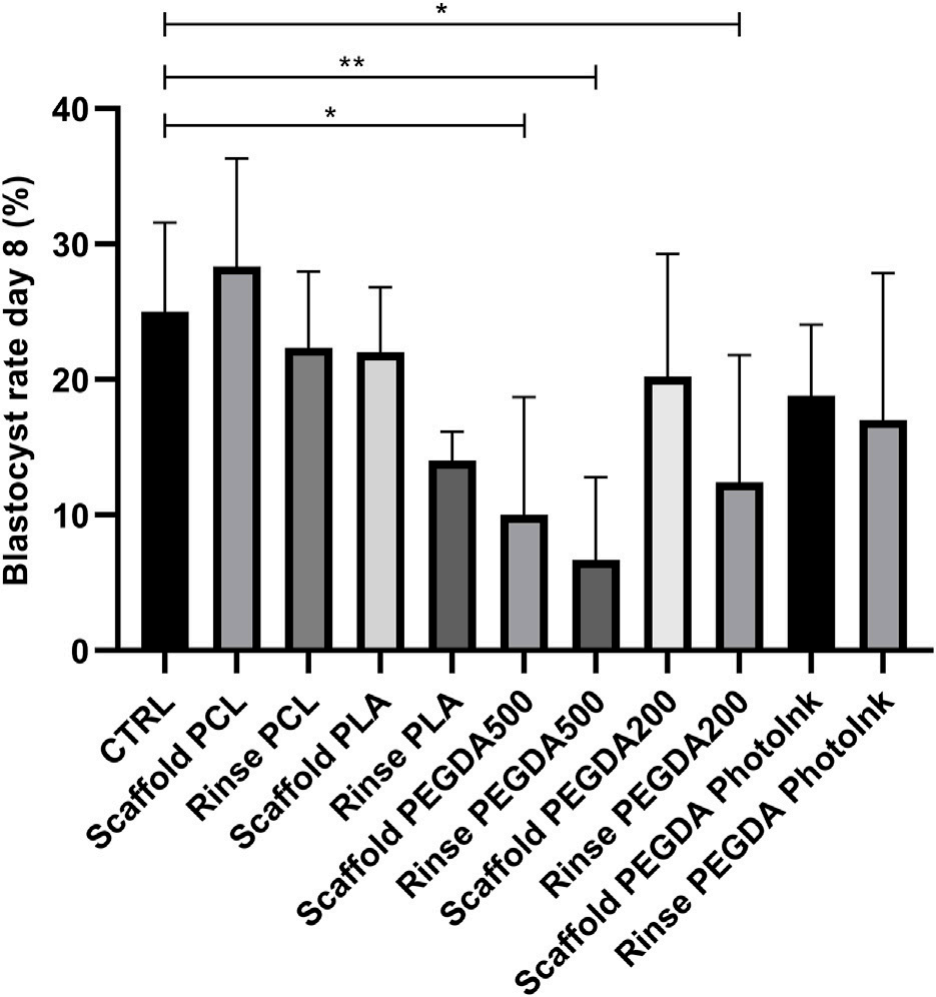
Effect of different materials scaffolds on blastocyst rate at day 8. The histograms show the blastocyst rate at day 8 when the IVF was performed with unconditioned medium (CTRL), with medium conditioned by each material (rinse groups) or when different scaffolds were present (scaffold groups). The data shows significant differences (*p* < 0.05) in CTRL vs. Scaffold PEGDA500, CTRL vs. Rinse PEGDA500 and CTRL vs. Rinse PEGDA200. The data are presented as mean of 11 independent experiments. Data were analyzed using the Dunnett’s test. *p < 0.05, **p < 0.01 versus control.

Blastocyst rate at day 8 were significantly lower in rinse PEGDA500 (6% ± 6%), scaffold PEGDA500 (10% ± 9%), and rinse PEGDA200 (12% ± 9%) groups vs. CTRL group (25% ± 6%). On the scaffold’s groups, we had not statistical differences (*p* > 0.05) for blastocyst yield in the scaffold PCL group with 29% ± 8%, while the scaffold PLA (14% ± 2%), scaffold PEGDA200 (20% ± 9%) and scaffold PhotoInk (19% ± 5%) groups showed similar blastocyst rates. In addition, rinse groups did not present significant differences vs. the CTRL, being the blastocyst rates 22% ± 6% for rinse PCL, 14% ± 2% for rinse PLA, and 17% ± 11% for rinse PEGDA PhotoInk (Figure 4).

### 3.4 Principal component analysis of the different materials considering all variables studied

**FIGURE 5:**
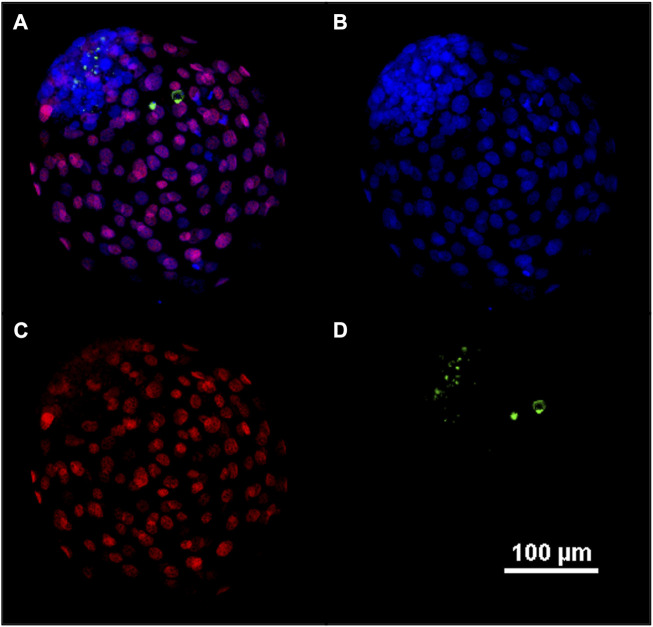
Representative confocal image of blastocyst at day 8. Fluorescent image of differential apoptotic staining **(A–D)**. At day 8, bovine blastocysts were fixed, dyed with Hoechst 33342 for nuclei **(B)**, immune-stained for CDX2 for the trophectoderm **(C)**, and for active caspase-3 for the apoptosis **(D)**. In **(A)** an overlay **(B–D)** is provided.

**FIGURE 6:**
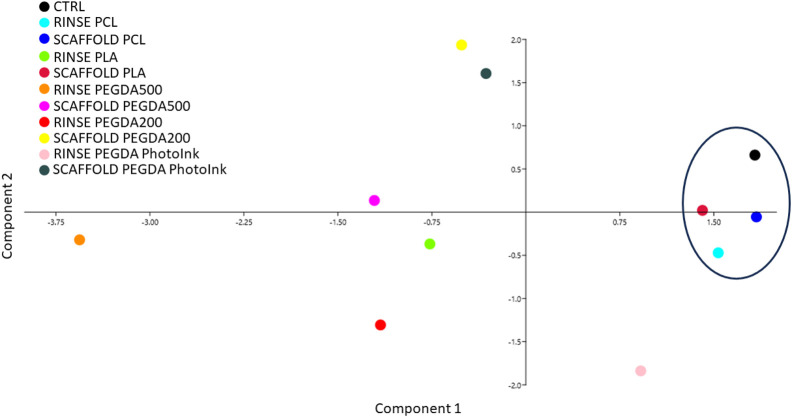
Principal component analysis performed by assessing the different parameters studied (Cleavage, blastocyst rate at days 7 and 8, TCN, ICM/TE and ACR). Principal component analysis shows no separation among groups. However, we observed that the closest groups to control are scaffold PLA, scaffold PCL and rinse PCL.

The total cell number, the trophectoderm and the apoptosis were evaluated under fluorescence microscopy (Figure 5) and the ICM/TE ratio and ACR were calculated. Since we studied several biological factors (cleavage, blastocyst rate at day 7 and 8, TCN, ICM/TE ratio and ACR), we used the Principal Component Analysis (PCA) as a multivariable analysis to simplify the data analysis and interpretation by reducing the complexity (Jollife and Cadima, 2016a). This analysis allows to reduce the amount of information needed since the system works with more compact representation of the data by retaining the relevant information and highlighting the underlying patterns and structures (Jollife and Cadima, 2016b) PCA showed how the scaffold PCL, rinse PCL, scaffold PLA and control groups were more similar among them than to the other groups (scaffold PEGDA500, rinse PEGDA500, rinse PEGDA200, scaffold PEGDA200, rinse PLA, rinse PhotoInk and scaffold PhotoInk) (Figure 6).

## 4 Discussion

In the present study we performed a bovine embryo assay to evaluate the potential toxicity of PLA, PCL, PEGDA500, PEGDA200, and PEGDA PhotoInk biomaterials in the embryo IVP during IVF. In addition, we tested not only different materials but also different printing methods, using for each material the most suitable method for the scaffold construction needs. We chose this animal model since it has already been used for embryo assay (Ieda et al., 2018) and it represents a valuable model for IVF improvement trials (Ménézo and Hérubel, 2002). It is also well-known that IVP produces suboptimal embryos with a lower yield of blastocysts and lower developmental capacity than their *in vivo* counterparts (Heras et al., 2016; Canovas et al., 2017; Ferraz et al., 2018b).

The first step in producing a device that could improve the IVP is the choice of biomaterial. All the materials we propose have been used in cell culture and have shown a good biocompatibility (Eslahi et al., 2013; Biagini et al., 2021; Di Berardino et al., 2022; Testore et al., 2022). However, as the cytotoxicity of the materials such as PLA (Biagini et al., 2021) could be different according to the cell types, the biocompatibility should be tested in regard to gametes, zygotes and embryos.

Our results suggest that the only material that has toxic effects is PEGDA500. This material had detrimental effect on bovine embryo development, promoting lower cleavage and lower blastocyst rate at day 7 and 8. This is an unexpected effect since PEGDA hydrogels have been suggested as effective candidates to carry out studies for embryogenesis and organogenesis due to their low cost, high reproducibility, and ease fabrication (Hribar et al., 2015). This is not the first time that biomaterials have shown unexpected negative effects when in contact with embryos. MacDonald et al. (2016) showed that VisiJet Crystal material (belonging to the strictest class for plastic biocompatibility) had a detrimental effect on zebrafish embryos (MacDonald et al., 2016). The materials E-shell200 and E-shell300 have also shown a deleterious effect on bovine embryo development, even having been considered biocompatible according to ISO 10993 (Ferraz et al., 2018a). Furthermore, we must take into consideration the eventuality that our materials might not be exactly the same in chemistry as those used in previously works, since the full chemical composition may vary from one company to another. Another plausible factor could be the fact that in the 3D printed scaffolds could be found some residues of toxic compounds that have been used to stabilize and print the devices. Indeed, several studies have observed that different chemical species are leaked by 3D-printed scaffolds (Oskui et al., 2016; Ferraz et al., 2018a).

Additionally, we detected significant differences between the rinse group of PEGDA200 and CTRL group, but no differences when PEGDA200 scaffolds were compared to the controls. This result suggests that the PEGDA200 may require longer washing than the other PEGDA hydrogels, since this type of scaffold had no detrimental effects on embryo development during IVF after being washed for 24 h and rinsed for another 24 h. However, all these hydrogels might not be the best option for the IVF device manufacturing because they were very fragile, and their rupture could be a relevant inconvenience during sterilization and handling.

Conversely, neither PLA nor PCL have shown detrimental effects on cleavage and blastocyst rate parameters. The PLA synthetic polymer has been suggested as an optimal candidate for scaffold fabrication due to its high biocompatibility, low cost, and mechanical properties (Serra et al., 2013; Di Prima et al., 2016). To our knowledge, this is the first study testing those materials to support bovine IVF, showing high biocompatibility. This is an expected result since both PLA and PCL biomaterials have been used in an emerging field called REPROTEN, the discipline that applies tissue engineering to restore fertility (Amorim, 2017). It has been shown that PLA is a suitable material to create a nanofiber scaffold that enhance the *in vitro* cluster formation of mouse spermatogonia stem cells, allowing their proliferation and differentiation (Eslahi et al., 2013; Ghorbani et al., 2019). As well, PCL has been used to culture spermatogonia stem cells (Talebi et al., 2019; Ghorbani et al., 2022), obtaining the same successful results as PLA. Furthermore, recent works have used PCL scaffolds as devices to carry out folliculogenesis in sheep (Di Berardino et al., 2022) and pig (Liverani et al., 2019).

We observed worse results with the increase of the PEGDA material stiffness (PEGDA Photoink vs PEGDA200 vs. PEGDA500). Previously, it has been shown that the stiffness of different substrates can affect *in vitro* embryo development in mice (Kolahi et al., 2012), but in our case the lower efficiency could be due to the chemistry employed to promote higher stiffness of the material, since even when the scaffold is absent during IVF, the rinse groups showed lower efficiency. One possible explanation for the worse performance of the rinse groups could be that the scaffolds release toxic compounds during the rinse period culture, so when the scaffolds are used during IVF the release of these toxic chemicals is much lower or absent. However, to confirm this hypothesis mass spectrometry analysis should be performed.

Altogether, these data suggest that the materials printed with stereolithography (PEGDAs) are less biocompatible than extrusion-printed materials (PLA and PCL). On the other hand, the TCN, the ICM/TE ratio and the ACR are three important parameters of embryo quality and in recent years, several studies have shown that the rate of ICM/TE is a strong predictor of live birth (Ai et al., 2021; Sivanantham et al., 2022). When we analyzed all these parameters, the principal component analysis (PCA) showed that the embryos produced in presence of PLA and PCL scaffolds are the most comparable to the control group. Regarding the PCL biopolymer, the rinse and the scaffold groups are both closer to the control ones, in terms of the analyzed parameters. While a different situation using the PLA biomaterial has been observed, since the scaffold group exhibited similar behavior to the control, contrary to the rinse group of the same biopolymer. For this reason, we consider PCL as the most suitable material for *in vitro* bovine embryo production.

Considering that we have not identified any negative effects on bovine embryo development when PCL is present during IVF, its implementation in the construction of a device compatible with microfluidics systems becomes a promising possibility. The combination of these microfluidics systems with the above-mentioned devices could allow the creation of an *in vitro* model of the oviduct (Romar et al., 2019). This innovative application could have a significant impact on the research and understanding of sperm selection by mimicking rheotaxis, chemotaxis and thermotaxis (Pérez-Cerezales et al., 2018; Ramal-Sanchez et al., 2021), fertilization and early development processes, providing a controlled and reproducible environment for experimental studies, without jeopardizing early embryo development.

In conclusion, the utilization of PCL in the construction of an IVF device holds great promise for the improvement of ARTs in the near future. However, further research and development are necessary to test the biocompatibility with OECs, optimize the design and functionality of this PCL-based IVF devices, ensuring their long-term effectiveness and safety. Nonetheless, the outcomes of our study strongly support the potential of the PCL biomaterial and open the way for advancements in the field of ARTs.

## Data Availability

The original contributions presented in the study are included in the article/Supplementary Material, further inquiries can be directed to the corresponding author.
